# Validity and reliability of an accelerometer-based assessgame to quantify upper limb selective voluntary motor control

**DOI:** 10.1186/s12984-020-00717-y

**Published:** 2020-07-13

**Authors:** Jeffrey W. Keller, Annina Fahr, Julia Balzer, Jan Lieber, Hubertus J. A. van Hedel

**Affiliations:** 1 Swiss Children’s Rehab, University Children’s Hospital Zurich, Affoltern am Albis, Switzerland; 2 Children’s Research Center, University Children’s Hospital Zurich, Zurich, Switzerland; 3 Doctoral Program Clinical Science, Faculty of Medicine, University of Zurich, Zurich, Switzerland; 4 Centre for Health, Activity and Rehabilitation Research, Queen Margaret University, Edinburgh, Scotland

**Keywords:** Assessgame, Psychometric properties, Selective voluntary motor control, Accelerometer sensors, Inertial measurement units, Upper extremities, Upper motor neuron lesions, Interactive computer play

## Abstract

**Introduction:**

Current clinical assessments measure selective voluntary motor control (SVMC) on an ordinal scale. We introduce a playful, interval-scaled method to assess SVMC in children with brain lesions and evaluate its validity and reliability.

**Methods:**

Thirty-one neurologically intact children (median [1st-3rd quartile]: 11.6 years [8.5–13.9]) and 33 patients (12.2 years [8.8–14.9]) affected by upper motor neuron lesions with mild to moderate impairments participated. Using accelerometers, they played a movement tracking game (assessgame) with isolated joint movements (shoulder, elbow, lower arm [pro−/supination], wrist, and fingers), yielding an accuracy score. Involuntary movements were recorded simultaneously and resulted in an involuntary movement score. Both scores were normalized to the performance of 33 neurologically intact adults (32.5 years [27.9; 38.3]), which represented physiological movement patterns.

We correlated the assessgame outcomes with the Manual Ability Classification System, Selective Control of the Upper Extremity Scale, and a therapist rating of involuntary movements. Furthermore, a robust ANCOVA was performed with age as covariate, comparing patients to their healthy peers at the age levels of 7.5, 9, 10.5, 12, and 15 years.

Intraclass correlation coefficients and smallest real differences indicated relative and absolute reliability.

**Results:**

Correlations (Kendall/Spearman) for the accuracy score were τ = 0.29 (*p* = 0.035; Manual Ability Classification System), ρ = − 0.37 (p = 0.035; Selective Control of the Upper Extremity Scale), and ρ = 0.64 (*p* < 0.001; therapist rating). Correlations for the involuntary movement metric were τ = 0.37 (*p* = 0.008), ρ = − 0.55 (*p* = 0.001), and ρ = 0.79 (*p* < 0.001), respectively. The robust ANCOVAs revealed that patients performed significantly poorer than their healthy peers in both outcomes and at all age levels except for the dominant/less affected arm, where the youngest age group did not differ significantly. Robust intraclass correlation coefficients and smallest real differences were 0.80 and 1.02 (46% of median patient score) for the accuracy and 0.92 and 2.55 (58%) for involuntary movements, respectively.

**Conclusion:**

While this novel assessgame is valid, the reliability might need to be improved. Further studies are needed to determine whether the assessgame is sensitive enough to detect changes in SVMC after a surgical or therapeutic intervention.

## Introduction

Patients with upper motor neuron lesions, for example such affected by cerebral palsy (CP), traumatic brain injury, or stroke, often exhibit multiple symptoms contributing to their disability. These symptoms can be classified as either being positive or negative motor signs. Positive signs are characterized by an increased frequency and amplitude of involuntary muscle activation, whilst patients with negative motor signs exhibit insufficient muscle activity or an impaired control [1]. While negative motor signs might contribute more to a child’s disability [2, 3], they are also more difficult to measure [1]. The importance of these negative motor signs, especially selective voluntary motor control (SVMC), as a predictor of gross motor function has been demonstrated in children [4, 5].

SVMC has been defined as the *“ability to isolate the activation of muscles in a selected pattern in response to demands of a voluntary posture or movement”* [1]. SVMC develops during childhood and might decline later in life. For example, neurologically intact children (NIC) up to the age of 10 years can display mirror movements when performing tasks with the upper extremities [6]. Also in adults aged 50–80 years, mirror movements have been observed [7]. The manifestation of reduced SVMC in the form of observable involuntary movements, however, is task dependent [8]. Hence, it is essential to interpret results of patients with neurological disorders performing SVMC assessments in the context of the performance of not only young, neurologically intact adults (NIA) but also age-matched healthy peers.

With regard to the upper extremity movements of patients, reduced SMVC has been shown to impact activities of daily living in children with CP [9, 10]. However, a clinical tool to solely assess reduced SVMC of the upper extremities has only recently been available [11]. The Selective Control of the Upper Extremity Scale (SCUES) [12] evaluates SVMC by letting patients perform isolated joint movements three times and assessing simultaneously occurring mirror movements (movements of the contralateral joint), movements of the trunk or any other joint apart from target joint. In addition, if the patient displays an active range of motion that is smaller than the passive one, SVMC is also rated as impaired. Each target joint is tested and rated on a 4-point, ordinal scale ranging from 0, no selective motor control, to 3, normal selective motor control.

Despite the SCUES being valuable to rate SVMC without much equipment, a drawback of the ordinal scale might be a lack of sensitivity to detect change occurring after interventions. Furthermore, rating the extent of involuntary movements and the active range of motion the target joint can be somewhat subjective.

Here we evaluate the validity and reliability of a novel, interval-scaled assessment game (assessgame) that uses accelerometers to quantify SVMC of the upper extremities objectively.

## Methods

### Participants

The aim was to recruit 30 NIA between the age of 18 to 50 years, by convenience sampling, as well as 30 NIC, aged 6 to 18 years by quota sampling. Because involuntary movements are reported more frequently for younger age categories [6, 13, 14], we aimed to recruit more participants in the age range of 6 to 10 years.

Patients with a diagnosed upper motor neuron lesion, aged between 6 and 18 years, with the ability to understand and follow simple instructions and sit upright for 1 h with backrest support, were included. Exclusion criteria were the treatment with Botulinum toxin or any surgical intervention of the upper extremities in the past 6 months. We recruited in- and outpatients of the Swiss Children’s Rehab. Affoltern am Albis by convenience sampling.

All participants were characterized by using descriptors of age, gender, and handedness, defined by which hand is used to write/draw. We additionally recorded the diagnosis and more affected (or non-dominant) hand, determined by an occupational therapist.

All methods were in accordance with the necessary guidelines and approved by the ethical committee of the canton of Zurich, Switzerland (PB_2016_01843). Either the participant and/or the legal guardian gave written informed consent.

### Assessgame

**Fig. 1:**
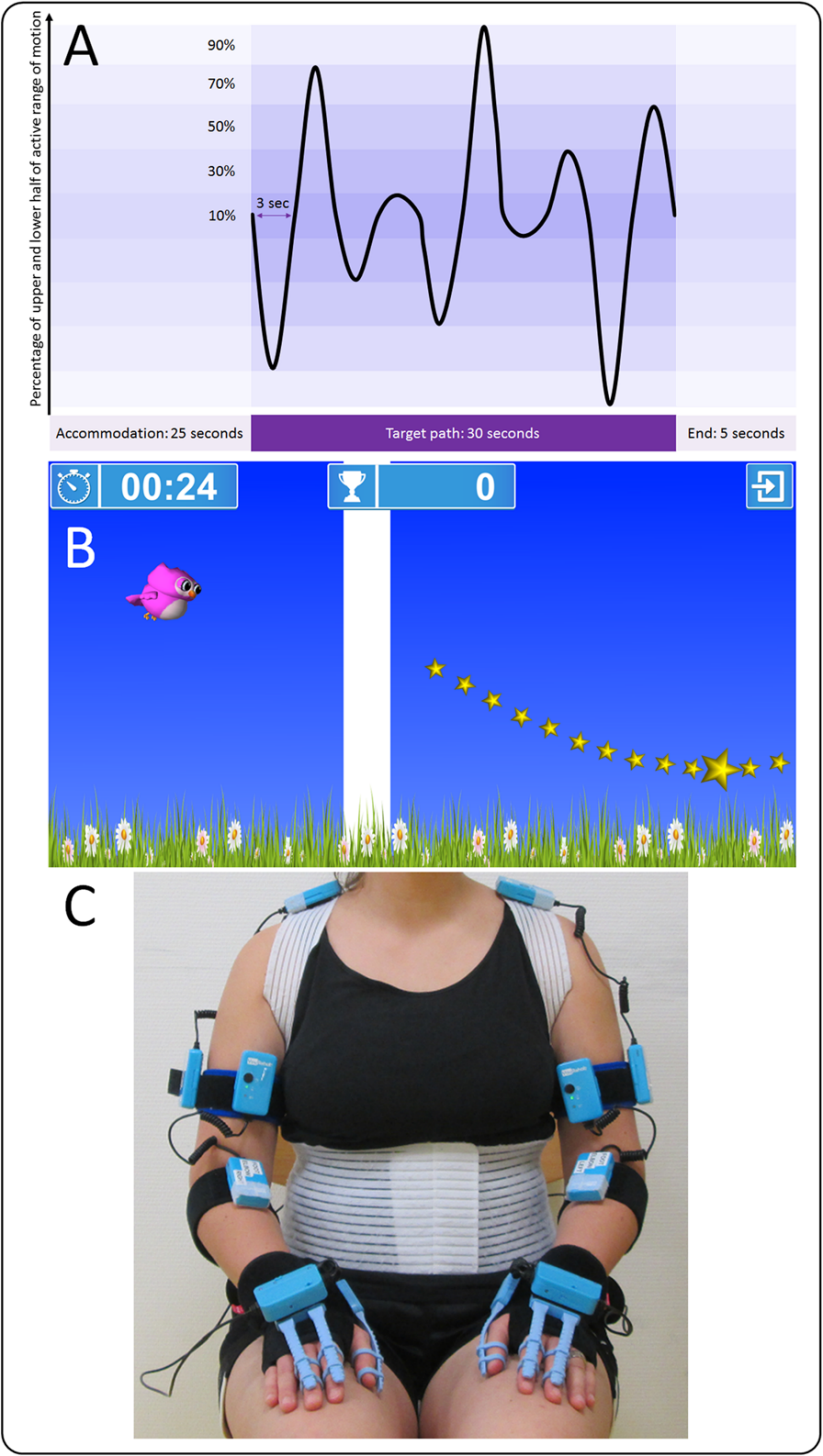
Assessgame elements and sensor placement. **a** Predefined target path. After a 25 s accommodation phase players follow the target path for 30 s challenging them within 90% of their active range of motion. The end phase of 5 s is not analyzed. **b** Avatar owl crossing the line from accommodation phase to target path. Steering the owl, the goal is to follow the star-studded target path as accurately as possible without any involuntary movements. **c** Placement of the accelerometer sensors proximal and distal of all target joints. Abbreviations: sec = seconds

The assessgame was created in collaboration with Reha-Stim Medtech AG (previously YouRehab, Schlieren, Switzerland). Its technical features and in depth description can be found in a separate methods paper [15]. In short, the assessgame uses accelerometers and bend sensors (cyber-gloves for fingers) to capture target joint movements and (potentially) simultaneously occurring involuntary movements. The goal is to move the target joint in an isolated manner to steer the avatar on a star-studded, predefined path. The path challenges players within 90% of their active range of motion (Fig. 1a), calibrated before each movement. Accelerometer sensors were applied proximally (reference sensor) and distally of the joints (Fig. 1c) to ensure that compensatory movements had no influence on the avatars path.

**Fig. 2:**
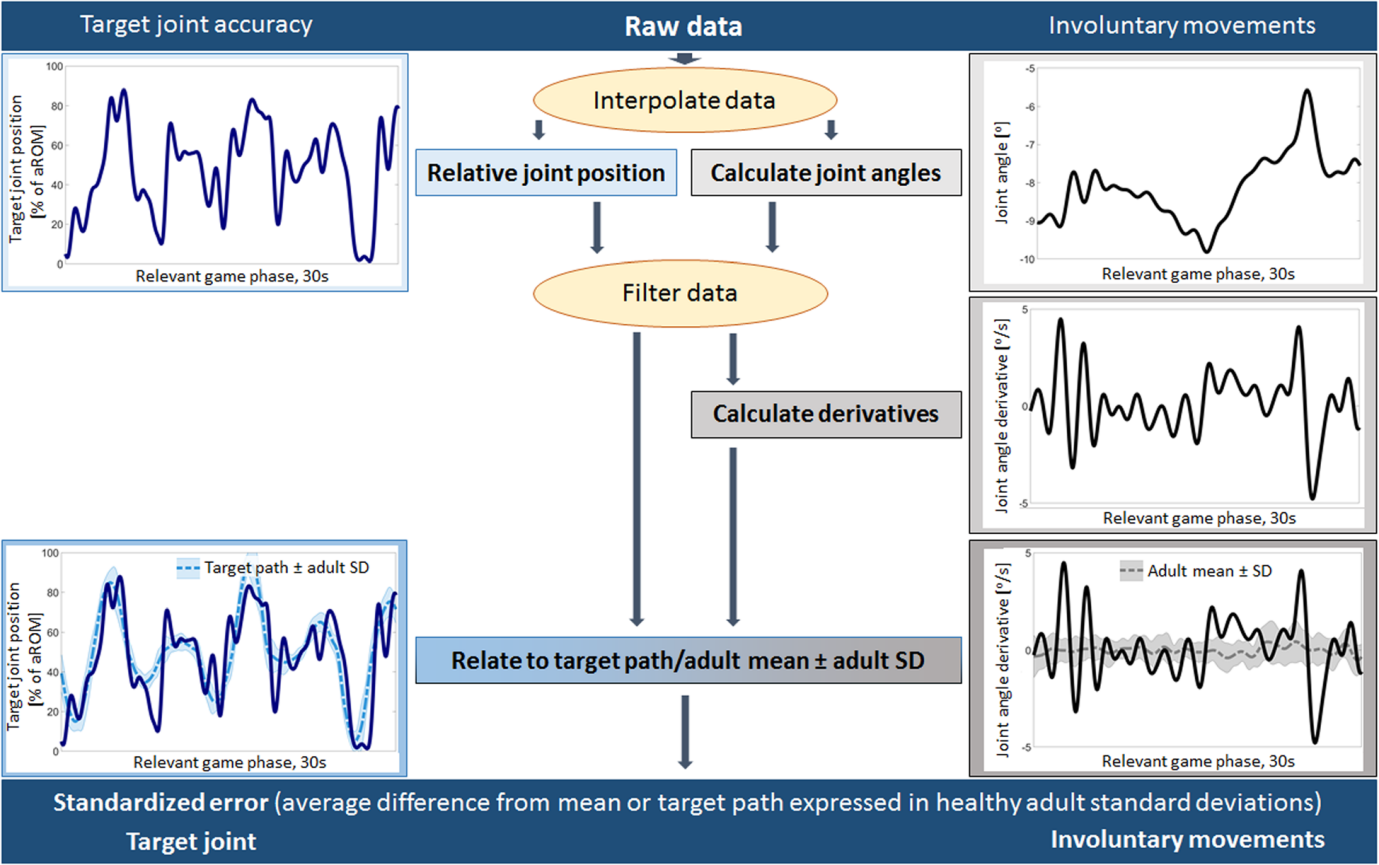
Visualization of the assessgame’s data analysis steps. The assessgame splits selective voluntary motor control (SVMC) into target joint accuracy and involuntary movements. We visualized the algorithm analyzing the raw accelerometer data resulting in standardized error scores for both outcome metrics. For the target joint, the numbers between 0 and 100 reflect the percentage joint position relative to the calibrated active range of motion. The involuntary movements were analyzed by first calculating the actual joint angle and then the derivative to quantify changes in position. This was done so that patients who were unable to maintain the starting position were not penalized. Finally, the standardized error expresses how many adult standard deviations the player was away from either the target path or the adult mean (involuntary movements) on average. The data were filtered using a 6th order Butterworth zero-phase low-pass filter with a cutoff frequency of 1.5 Hz (normalized cutoff frequency of 0.045). Reprinted from 'First validation of a novel assessgame quantifying selective voluntary motor control in children with upper motor neuron lesions' [15]

The assessgame provides two outcome measures, one for the accuracy of the target joint movement and one combined value for the involuntary movements. Further information on the exact algorithm used to generate these outputs can also be found in the methods paper [15]. In summary (Fig. 2), for the target joint accuracy score, the avatar position was calculated relative to the calibrated active range of motion. The absolute difference between the avatar position and the predefined path was calculated and divided by the standard deviation of the NIA around the path. This latter step allows to interpret deviations from the predefined path in relation to the difficulty of the trajectory (as in more difficult sections of the path, the SD of the NIA will be larger. Finally, we calculated an average accuracy score of the target joint. The score for the involuntary movements was calculated similarly. From the accelerometer data, joint angles were calculated, which were used to calculate the change in joint angle per time unit (derivative). The derivatives of the NIA served as reference path for all participants. The absolute difference between the derivative of participant’s path and the reference path was then divided by the SD of the NIA around the reference path. The same procedure, albeit without calculating joint angles, was used for the finger data generated by the bend sensors. The average of each joint was then averaged with the rest of the joints resulting in the involuntary movement score.

### Set up and procedure

Participants performed the German version of the SCUES [16] before starting with the assessgame. Table and chair height were adjusted such that the participant’s hip, knees, ankles, and elbows were in a 90-degree angle.

Playing with the upper extremities, the avatar is steered by abducting and adducting the shoulder (with a 90-degree flexed elbow), flexing and extending the elbow (against gravity, vertical upper arm), wrist and fingers (both supported on a firm cushioning with pronated lower arms), and by pronating/supinating the lower arm on a table. The sensors were attached with Velcro straps, the positions are displayed in Fig. 1c. When playing with the shoulder and elbow joint, the participant’s arms were unsupported, for the other joints the table and cushioning were used, allowing for easier movement execution and minimizing the impact of the sensors on performance.

After receiving verbal instructions on the game (e.g., follow the star-studded path and only move the target joint), participants played three trial rounds, familiarizing with the different steering mechanisms, using the following joints: fingers, forearm, and either elbow or shoulder. Since pilot-testing had revealed that playing the game with pronation/supination of the lower arm was less intuitive, participants were asked to train that movement in the trial rounds. Playing with the fingers familiarized the participant with the cyber-gloves’ bend sensors. Finally, the participants could decide if they either preferred playing with the elbow or the shoulder joint because the steering mechanisms are the same and analogous to the wrist. After three trial rounds, the participant played with all 10 target joints in a randomized order to account for learning effects. While the participant was playing, the therapist noted any involuntary movements occurring. The possible descriptors were defined as mirror movements (movements in the contralateral, homologous joint), movements of any other joints, and of the trunk. The therapist could note any combination of descriptors. Administering the assessment took around 25 to 35 min, including the breaks that were allowed to avoid fatigue.

To evaluate the reliability of the measurements, the assessgame was repeated 1 to 3 days later with inpatients and 7 days after the initial measurement with outpatients.

### Measurement tools

The assessgame measures target joint accuracy and involuntary movements occurring. Both metrics are measured on an interval scale where a score of zero is the theoretically possible perfect score and increasing values indicate a greater deviation from the target paths.

The involuntary movements that the therapist noted (henceforth called therapist opinion) were converted into a sum score for every target joint that was played. The score simply summed all descriptors the therapist noted. Therefore, a score of zero meant that the participant showed no involuntary movements while performing the target joint movement and a score of 3 indicated that all penalized involuntary movements were displayed (mirror and trunk movements and also movements of any additional joints).

The Manual Ability Classification System (MACS) [17] was validated in children with CP and classifies how they handle objects in daily activities. Patients with level 1 handle objects easily and successfully whereas level 5 indicates that patients do not handle objects at all [17]. Medical professionals in our rehabilitation center routinely assess the MACS level in children with CP. For this study, they also classified children with other diagnoses.

The SCUES [12] was also validated in children with CP and evaluates SVMC on a four-point ordinal scale, for each target joint separately. A score of zero means that there is no observable SVMC and a score of 3 that the participant performs the desired movement over the entire range of motion without displaying any involuntary movements. The SCUES tests the shoulder, elbow, lower arm, wrist, fingers analogous to the assessgame with one exception, elbow flexion and extension are measured while the arm is in a horizontal position, thus mitigating the effect gravity has on performance.

Both the MACS and SCUES have not been validated in patients with upper motor neuron lesions other than CP. Therefore, the MACS and SCUES values of patients with other diagnoses should be considered to approximate the handling of objects in daily life and SVMC, respectively.

### Statistical analysis

The statistical analysis was performed in R (version 3.5.1) [18] using the additional packages boot (v 1.3–20) [19], ICC (v 2.3.0) [20], mice (v 3.3.0) [21], and WRS2 (v 0.10–0) [22].

Before statistically analyzing the data, missing data points due to sensor errors were imputed with the mean value resulting from multiple imputation by chained equation. For detailed information we refer to our methods paper [15].

Convergent validity was tested by correlating the accuracy and involuntary movement metrics of the assessgame with the MACS, SCUES and therapist opinion for each individual joint, for the average score for the less/more affected side, and the combined average of all joints. Kendall’s tau-b [23] was chosen as correlation coefficient because it is specifically designed to handle ties in the data, of which there had to be many by nature of the few levels the ordinal scales provide. Additionally to the tau-b, we calculated Spearman’s rank correlation coefficient for the average scores per limb, because the sum scores were expected to show more dispersion, hence warranting both correlation types. For the total involuntary movement score, we expected a high, positive Spearman correlation (0.7 ≤ ρ < 0.9) with the therapist opinion, a moderate, negative correlation (− 0.5 ≥ ρ > − 0.7) with the SCUES, and a low, positive correlation (0.3 ≤ ρ < 0.5) with the MACS. Total target joint accuracy correlations were expected to be moderate and positive for the therapist opinion, low and negative for the SCUES and low and positive for the MACS.

Discriminative validity was tested by comparing the NIC to the patient group for both assessgame metrics. This was done for the average scores of the less/more affected sides and the combined average scores of all joints. A robust, bootstrapped ANCOVA, as described by Mair and Wilcox [24], was used, entering age as a covariate. This method of analysis allows for robust comparisons at distinct levels of the covariate, in our case at the age levels 7.5, 9, 10.5, 12, and 15 years. The number of bootstrap samples was set to 2′000 and the data were not trimmed. Bootstrapped 95% confidence intervals were adjusted for the multiple comparison points. The span parameter (defining model flexibility) was set as low as possible (greater flexibility) but such that the group sizes at the comparison levels were at least 12, as suggested by Mair and Wilcox [24].

Relative reliability was evaluated by calculating two-way random effects, absolute agreement, single/measurement intraclass correlation coefficients (ICCs) according to Koo and Li [25]. ICCs were classified as poor (ICC < 0.5), moderate (0.5 ≤ ICC < 0.75), good (0.75 ≤ ICC < 0.9), excellent (ICC ≥ 0.9) [25], and considered acceptable when above 0.75. To account for non-normally distributed data, we used the bias-corrected and accelerated bootstrap method [26] to construct 95% confidence intervals. The number of bootstrap samples taken was 1′000.

Due to the heterogeneous patient population, we used a conservative approach to determine the smallest real difference as a measure of absolute reliability. We divided the difference between the 97.5 percentile and 2.5 percentile by 2 and bootstrapped this metric 2′000 times. The upper limit was then taken as a robust estimate of absolute reliability (resembling the Bland-Altman approach for normally distributed data).

## Results

A total of 33 NIA and 31 NIC participated and served as reference groups. In total 8 patients of 41 that gave informed consent dropped out of the study, 4 of them due to the severity of their disability and 4 due to compliance issues. Of the remaining 33 patients, only 23 were available for a second (reliability) assessment. Their characteristics are listed in Table 1. Patients participating in the validity part were similar to the NIC with regard to median age and distribution, but gender proportions stand out as dissimilar. For all children with upper motor neuron lesions except one, the dominant arm was also the less affected one. Twenty patients (61%) had a diagnosis where one side was acknowledged as being more affected.

**Table 1:** Participant group characteristics and assessment scores

Number of participants	NIA	NIC	Patients (validity)	Patients (reliability)
	33	31	33	23
Age^M^	32.5 [27.9; 38.3]	11.5 [8.5; 13.9]	12.2 [8.8; 14.9]	9.9 [8.7; 15.0]
Gender: female	18 (55%)	16 (52%)	11 (33%)	9 (39%)
Diagnosis				
Cerebral palsy			22 (67%)	15 (65%)
Stroke			8 (24%)	6 (26%)
Traumatic brain injury			2 (6%)	2 (9%)
Encephalitis			1 (3%)	0 (0%)
MACS				
1			7 (21%)	3 (13%)
2			12 (36%)	11 (48%)
3			13 (40%)	8 (35%)
4			1 (3%)	1 (4%)
Total SCUES^M^				
Dominant/less affected			13.0 [11.0; 14.0]	13.0 [11.5; 14.5]
Non-dominant/affected			9.0 [7.0; 13.0]	8.0 [6.5; 13.0]
Both sides			21.0 [18.0; 26.0]	21.0 [18.0; 26.0]
Avg. therapist opinion^M^				
Dominant/less affected			0.8 [0.6; 1.2]	0.8 [0.6; 1.1]
Non-dominant/affected			1.0 [0.8; 1.6]	1.0 [0.7; 1.7]
Both sides			1.0 [0.7; 1.3]	1.0 [0.6; 1.3]
Avg. AG target joint accuracy^M^				
Dominant/less affected	0.7 [0.7; 0.8]	1.1 [0.8; 1.5]	1.9 [1.7; 2.8]	2.0 [1.8; 2.8]
Non-dominant/affected	0.7 [0.7; 0.9]	1.0 [0.8; 1.4]	2.5 [1.9; 3.3]	2.6 [2.1; 3.1]
Both sides	0.7 [0.7; 0.9]	1.1 [0.8; 1.4]	2.4 [1.6; 2.8]	2.4 [2.0; 2.8]
Avg. AG involuntary movements^M^				
Dominant/less affected	0.7 [0.7; 0.9]	0.9 [0.7; 1.6]	3.0 [1.6; 5.4]	3.8 [2.0; 6.1]
Non-dominant/affected	0.8 [0.6; 0.9]	0.8 [0.7; 1.6]	4.0 [1.9; 7.8]	4.6 [3.1; 8.2]
Both sides	0.7 [0.7; 0.9]	0.9 [0.7; 1.7]	3.6 [1.9; 6.0]	4.2 [2.4; 6.5]

*Abbreviations*: *NIA* neurologically intact adults, *NIC* neurologically intact children, *MACS* Manual Ability Classification System, *SCUES* Selective Control of the Upper Extremity Scale, *Avg*. average, *AG* assessgame, ^*M*^ median [1st; 3rd quartile]

### Convergent validity

In general, correlations of the assessgame metrics were stronger with therapist opinion than with the MACS and SCUES, especially for the involuntary movement metric, where they were high (Table 2). The MACS and the SCUES showed similar correlation coefficients and were small for the assessgame accuracy and moderate for the involuntary movement score. Furthermore, correlations were higher for the more affected arm compared to the less affected arm, for the averaged scores of all joints compared to the individual joints, and for the involuntary movement metric compared to the target joint accuracy.

**Table 2:** Convergent validity, relative and absolute reliability for individual joints and total scores of the assessgame

				MACS	SCUES	Therapist opinion	ICC (95%-CI)	SRD [1st; 2nd; 3rd quartile of patient scores]
Target joint accuracy	Less affected	shoulder		0.22	−0.23	0.28*	0.52 (0.29; 0.72)	1.95 [1.18; 1.91; 2.33]
		elbow		0.39**	−0.16	0.35*	0.36 (0.12; 0.61)	4.02 [1.51; 1.79; 2.75]
		lower arm		0.02	−0.04	0.28	0.82 (0.60; 0.95)	0.77 [1.47; 1.84; 2.34]
		wrist		0.25	−0.23	0.29*	0.63 (0.43; 0.76)	1.68 [1.26; 1.66; 2.39]
		fingers		0.22	−0.26	0.33*	0.58 (0.24; 0.84)	1.70 [1.71; 2.34; 2.85]
		mean		0.28*	−0.38**	0.40**	0.76 (0.63; 0.87)	1.13 [1.62; 1.96; 2.71]
			ρ	0.33	−0.49**	0.54**		
	More affected	shoulder		0.32*	−0.20	0.31*	0.46 (0.18; 0.65)	1.63 [1.41; 1.95; 2;46]
		elbow		0.45**	−0.29*	0.42**	0.61 (0.31; 0.88)	2.94 [1.88; 2.47; 3.40]
		lower arm		0.49**	−0.39*	0.65***	0.73 (0.41; 0.90)	1.52 [1.70; 2.32; 3.48]
		wrist		0.34*	−0.52***	0.27	0.79 (0.56; 0.91)	1.96 [1.40; 2.40; 3.25]
		fingers		0.22	−0.27	0.26	0.57 (0.10; 0.80)	1.87 [1.77; 2.52; 3.46]
		mean		0.31*	−0.26*	0.52***	0.85 (0.71; 0.93)	0.99 [1.89; 2.38; 2.98]
			ρ	0.39*	−0.36*	0.62***		
	Both sides	mean		0.29*	−0.26*	0.50***	0.80 (0.62; 0.91)	1.02 [1.68; 2.24; 2.66]
			ρ	0.37*	−0.37*	0.64***		
Involuntary movements	Less affected	shoulder		0.35*	−0.17	0.47**	0.70 (0.50; 0.84)	3.91 [1.63; 3.66; 5.70]
		elbow		0.39**	−0.31*	0.59***	0.70 (0.38; 0.88)	7.92 [1.77; 3.86; 6.27]
		lower arm		0.25	−0.25	0.32*	0.60 (0.34; 0.86)	5.04 [1.54; 3.10; 4.40]
		wrist		0.23	−0.18	0.39**	0.48 (0.24; 0.79)	8.53 [1.61; 3.01; 4.70]
		fingers		0.20	−0.22	0.48**	0.68 (0.15; 0.94)	7.83 [1.57; 2.80; 5.20]
		mean		0.33*	−0.38**	0.47***	0.84 (0.55; 0.92)	4.43 [1.73; 3.62; 5.96]
			ρ	0.40*	−0.52**	0.62***		
	More affected	shoulder		0.29*	−0.21	0.43**	0.81 (0.71; 0.88)	3.58 [2.48; 3.69; 6.20]
		elbow		0.34*	−0.21	0.51**	0.49 (0.10; 0.78)	10.44 [2.23; 4.48; 7.70]
		lower arm		0.49**	−0.53**	0.66***	0.54 (0.23; 0.89)	11.72 [2.45; 4.00; 7.79]
		wrist		0.35*	−0.44**	0.50**	0.65 (0.41; 0.94)	9.39 [1.33; 3.45; 5.01]
		fingers		0.44**	−0.43**	0.60***	0.48 (0.21; 0.77)	16.68 [2.04; 3.56; 6.75]
		mean		0.37**	−0.32*	0.61***	0.84 (0.70; 0.93)	4.54 [2.68; 4.68; 7.72]
			ρ	0.46**	−0.49**	0.77***		
	Both arms	mean		0.37**	− 0.37**	0.64***	0.92 (0.82; 0.97)	2.55 [2.42; 4.38; 6.34]
			ρ	0.45**	−0.55**	0.79***		

All correlation coefficients are Kendall’s Tau except for the rows marked with ρ, which are Spearman’s correlation coefficients. The *p*-values of the correlation coefficients are indicated by asterisks: *(0.05 > *p* ≥ 0.01), ** (0.01 > *p* ≥ 0.001), and *** (*p* < 0.001)

*Abbreviations*: *MACS* Manual Ability Classification System, *SCUES* Selective Control of the Upper Extremity Scale, *ICC* intraclass correlation coefficient, *95%-CI* bias-corrected and accelerated, bootstrapped 95% confidence interval, *SRD* smallest real difference

### Discriminative validity

**Fig. 3:**
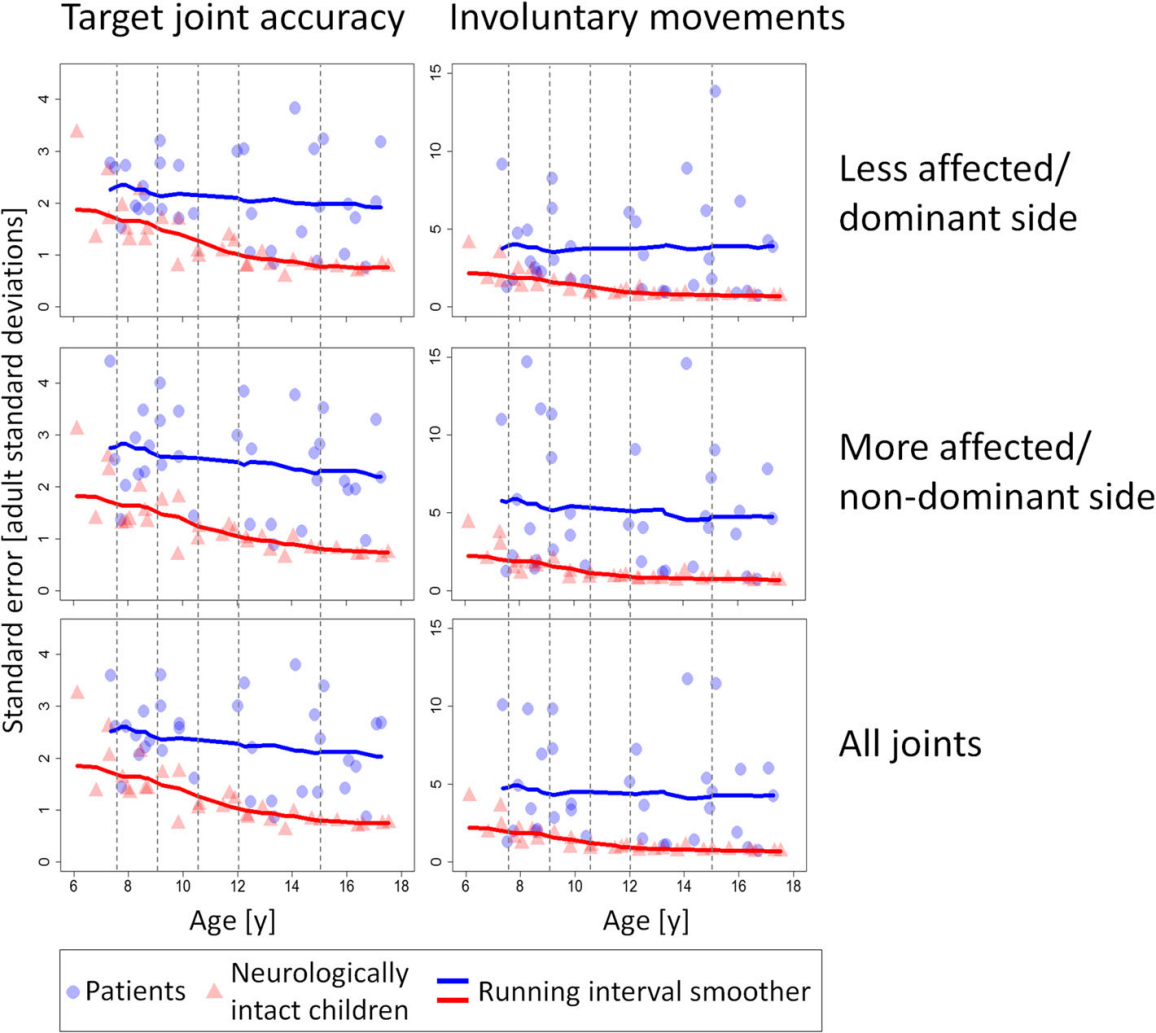
Assessgame outcomes for patients and healthy peers by age. Robust ANCOVAs using running interval smoothers (means without trimming) compared patients to their peers at predetermined levels (dashed lines) of the covariate age (see Table 3 for exact numbers). The span parameter was set to 1 for the patient group and 0.7 for their peers

The results for the discriminative validity tests can be found in Table 3. After correcting for multiple testing within each metric and side, the groups differed significantly at all levels, except for the young age categories of the less affected/dominant side. Figure 3 displays the results across all levels of the covariate. Both patients and NIC improve with age. The slope of improvement, however, was less pronounced in patients. The involuntary movement score for the patients’ less affected side even worsens slightly with age.

**Table 3:** Differences between patients and healthy peers for both assessgame metrics at different age levels

	Age in years	Difference between groups at predetermined covariate levels					
		Target joint accuracy			Involuntary movements		
		Estimate	95%-CI		Estimate	95%-CI	
Less affected/ dominant side	7.5	0.56	−0.09	1.21	1.91	−0.23	4.05
	9	0.55	−0.06	1.16	1.85	0.01	3.69
	10.5	0.89	0.34	1.43	2.48	0.77	4.18
	12	1.08	0.58	1.57	2.80	0.95	4.65
	15	1.21	0.53	1.89	3.16	0.47	5.86
More affected/ non-dominant side	7.5	1.04	0.28	1.81	3.66	0.02	7.30
	9	1.02	0.31	1.74	3.44	0.41	6.48
	10.5	1.31	0.73	1.90	4.18	1.36	7.00
	12	1.41	0.90	1.91	4.17	1.73	6.62
	15	1.49	0.85	2.13	4.01	1.12	6.80
All joints	7.5	0.81	0.16	1.46	2.75	0.14	5.37
	9	0.79	0.17	1.42	2.61	0.38	4.86
	10.5	1.10	0.57	1.63	3.30	1.20	5.39
	12	1.24	0.76	1.71	3.45	1.52	5.38
	15	1.34	0.71	1.96	3.55	1.02	6.08

Results of the robust ANCOVAs comparing patients to their peers at predetermined covariate levels for both assessgame metrics with bootstrapped 95% confidence intervals. The span parameter was set to 1 for the patient group and 0.7 for their peers. *Abbreviation*: *95%-CI* bootstrapped 95% confidence interval, corrected for multiple testing

### Reliability

Relative and absolute reliability are displayed in Table 2. The ICCs ranged from good to excellent for all averaged values, whereas the ICCs for the individual joints are mostly moderate. Sporadically some individual joints had poor or good relative reliability. Smallest real differences in relation to the median performances were smaller for the accuracy score than for the involuntary movement score and larger for the more affected arm than the less affected one. For the individual joints of the involuntary movement score, smallest real differences were mostly larger than the median of the patients’ scores. For the mean scores, the smallest real differences ranged from approximately 42 to 122% of the median of patient scores.

## Discussion

This study assessed the validity and reliability of a novel accelerometer-based assessgame. Convergent and discriminative validity results indicate that the assessgame is valid and can discriminate between patients with upper motor neuron lesions and NIC. Relative reliability was good to excellent for the averaged scores but only moderate for individual joints. Absolute reliability, however, expressed as smallest real difference, was somewhere in the range 42 to 122% of the median of patient scores indicating that the assessgame might not be as sensitive as predicted.

### Convergent validity

Correlations of the assessgame with the therapist opinion were clearly the highest, as was hypothesized. The obvious reason is that as an involuntary movement occurs, the therapist notes it. Correlation coefficients might even have been higher if frequency and intensity of the involuntary movements had been graded too, as is done for other assessments, for example the Zurich Neuromotor Assessment [27]. The correlations with the SCUES were lower compared to the therapist opinion. A possible explanation for that may be attention modulation. During the SCUES, patients are asked to focus specifically on suppressing involuntary movements. Patients even could receive a second or third try, if the assessor believes a better result is achievable. When playing the assessgame, patients were also asked to perform the target movement and suppress involuntary movements but are then left to play the game. Focusing on playing the game can be considered an external focus of attention, which may lead to playing without thinking about controlling involuntary movement. It has been shown in NIA [28] as well as NIC [29] that guiding attention to the involuntary movements (internal focus) leads to improved inhibition. The same can be said for children with CP albeit suppression was possible to a lesser extent [30]. Conversely, studies have demonstrated that computer games, in simplicity comparable to our assessgame, can have a distracting effect [31, 32], which might further divert attention from suppressing involuntary movements.

The fact that correlations are higher for the involuntary movement metric compared to the accuracy metric could mirror the fact that both the SCUES and the therapist opinion are designed to capture involuntary movements. The correlations are, however, not very far apart, indicating that patients who showed more involuntary movements while playing also followed the target path less accurately.

Another observable pattern is that correlations within the involuntary movement metric are greater for the non-dominant arm. It has been shown that children and adolescents affected by CP exhibit more mirror movements in their less affected hand, when performing movements with their more affected hand [30, 33]. Even though patients with stroke did not show increased EMG activation on the contralateral side, they did exhibit increased ipsilateral muscle activation [34]. In line with those results, Sukal et al. [35] found increased ipsilateral joint coupling for patients with hemiplegic CP. Patients in our study also exhibited more involuntary movements when playing with their more affected side (Fig. 3) and thus there was a clearer separation between scores, which might lead to higher rank correlations with therapist opinion.

### Discriminative validity

The fact that NIC improve in a more complex motor task whilst displaying less involuntary movements with older age has been demonstrated before [6, 36] and is associated with the maturation of the corticospinal tract [37]. Expanding on that, Rosenbaum et al. [38] found that children with more severe CP show a less pronounced improvement and an earlier leveling off in gross motor function, which is in line with what we see with the assessgame our results. These facts explain why the assessgame performance difference between patients and their healthy peers grows with age. The finding that the groups were not always significantly different at a young age highlights the importance of a reference group. A worse performance in such motor tasks and showing more involuntary movements may be physiological at younger ages.

### Reliability

Relative reliability of the averaged scores was in the range that was seen in other studies investigating SVMC [39, 40]. Keeping in mind that we chose a conservative way of estimating the smallest real difference, the resulting values still seemed rather large. In part, this might be due to factors such as motivation, time of day when testing, which was not standardized, and fatigue from therapy sessions. It needs to be determined whether this assessgame is sensitive enough to detect changes in SVMC after a surgical or therapeutic intervention.

### Methodological considerations

An important consideration of SVMC measures in general is that certain movements are performed against gravity which could also make strength a relevant factor. By letting patients play the game with their active range of motion and not their passive one, the idea was to minimize the influence of strength on the assessgame outcomes. This, however, needs to be confirmed with further research.

Furthermore, the pro−/supination movement could have been visualized differently. We decided to keep it consistent over all movements, letting the avatar be steered upwards and downwards. On the one hand, this ensured that participants did not have to be familiarized with multiple visualizations. On the other hand, this seemed unintuitive for most participants, which likely caused an additional cognitive demand when controlling the game with this movement.

Moreover, the heterogeneity of neurological conditions included in this study needs to be addressed. Even though the group is representative of the patient population in our rehabilitation center, the different conditions (e.g., congenital versus acquired brain lesions) might introduce more variability to the data. However, even a group as ‘specific’ as children with unilateral CP can show very different joint torque coupling patterns, depending on whether the brain injury occurred pre- or peri- or post-natal. The coupling patterns of children affected by post-natal injuries, for example, resemble those of adults with stroke [35]. This indicates that even children with the same diagnosis may vary strongly in the patterns of involuntary movements they exhibit. Statistically, we used a robust bootstrapping technique to capture this as best as possible.

Lastly, the size and quantity of sensors should be reduced for future, clinical applications. It is conceivable to use considerably smaller and only 7 sensors to measure the same metrics.

## Conclusion

In conclusion, the presented assessgame is valid and shows good relative reliability. We expect that absolute reliability needs to be improved to sensitively measure changes stemming from interventions aiming to improve SVMC.

## Data Availability

Due to the small number of patients in our rehabilitation center and the heterogeneity of the study group, we can only provide the data upon reasonable request. For further information, please contact the corresponding author.

## Abbreviations

CP: Cerebral palsy
ICC: Intraclass correlation coefficient
MACS: Manual Ability Classification System
NIA: Neurologically intact adults
NIC: Neurologically intact children
SCUES: Selective Control of the Upper Extremity Scale
SRD: Smallest real difference
SVMC: Selective voluntary motor control
